# Diverticulitis in the Under-40 Population

**DOI:** 10.7759/cureus.56190

**Published:** 2024-03-14

**Authors:** Danisi Jacqueline, Kimberly Lince, Virgil K DeMario, Alexander Zarutskie, Shannon Cisse, Olutayo Sogunro

**Affiliations:** 1 Medicine, University of the Incarnate Word School of Osteopathic Medicine, San Antonio, USA; 2 Biomolecular Science, Central Connecticut State University, New Britain, USA; 3 Surgery, Hartford Healthcare, St. Vincent’s Medical Center, Bridgeport, USA

**Keywords:** emergent general surgery, general and laparoscopic surgery, medical management, hinchey classification, diverticulitis

## Abstract

Background: As obesity and lifestyle factors become more prevalent in younger populations, we are diagnosing and treating diverticulitis in younger patients. In this study, the demographics, risk factors for the development, and treatment of acute diverticulitis were assessed focusing on patients under the age of 40.

Methods: A retrospective review of the electronic medical records of a cohort of subjects diagnosed with diverticulitis was performed. Inclusion criteria included patients aged 18-40 who were treated for acute diverticulitis with or without any complications.

Results: Of the 109 patients, 40 patients required surgery, and 69 patients were managed conservatively. Analysis showed that the Hinchey classification (p<0.001) was the strongest predictor of treatment modality.

Conclusions: As the incidence of diverticulitis has increased in recent decades, so too has the frequency with which elective surgical procedures are performed as treatment. While these procedures are vital components in the management of diverticulitis, the majority of research comparing conservative versus surgical treatments has been done in patients over 50 years old. Although diverticulitis has been classically thought of as a disease of the elderly, it has become more prevalent in younger populations due to the rise of obesity and lifestyle modification in the under-40 population. Although the prevalence of treatment and diagnosis of acute diverticulitis in younger patients has risen, there is a paucity of data surrounding treatment protocols for diverticulitis in association with patient symptoms for patients under the age of 40 years old. Our study has found that there is a higher incidence of complications in diverticulitis in patients under the age of 40. Additionally, when considering the pattern of complication presentation in younger patients with complicated diverticulitis, surgical intervention might not be appropriate. The current treatment algorithm relates diverticulitis complications with surgical interventions. However, our data suggest that patients under the age of 40 presenting with abscesses or strictures may not need surgical intervention. This information could be particularly helpful in guiding physicians and younger patients in selecting the best choice of care and minimizing complications. Additionally, further research should help guide treatment protocol in this specific population of patients, as there is a lack of established guidelines pertaining to diverticulitis surrounding younger patients.

## Introduction

Diverticulosis is a disease of the gastrointestinal tract that results in outpouchings of mucosa, commonly in the sigmoid colon, that can lead to the development of inflammation, infection, and possible colonic perforation. Diverticulosis is thought to be a disease of aging, with some research showing as much as 60% of the population over the age of 60 contain diverticula [1]. When diverticula become inflamed or infected, this disease is now referred to as diverticulitis.

There are many theories as to why individuals develop diverticula, such as certain lifestyle habits, low-fiber diets, obesity, and genetics [2]. Although the development of diverticula is correlated with such lifestyle habits, the presence of diverticula does not mean that diverticulitis will develop [3]. There is a notable increase, however, in the documented occurrences of diverticulitis. Between 1998 and 2005, there was a 26% increase in the number of patients diagnosed with diverticulitis who were admitted for inpatient management or proceeded with elective surgery [4]. For patients under the age of 45, this increase was more than 70%. It is theorized that this is, in part, due to the increased presence of obesity within younger populations. Obesity within the United States was shown to increase from 12% in 1970 to 49% in 2007. Concurrently, patients diagnosed with diverticulitis also increased from 19% to 40% [5].

Patients with diverticulitis most commonly present with left lower quadrant abdominal pain, nausea, vomiting, diarrhea, fever, and/or constipation [6,7]. Diverticulitis is usually diagnosed clinically based on presenting symptoms and previous medical history. However, computed tomography, ultrasound, or, in some cases, diagnostic laparoscopy followed by exploratory laparotomy can be used to confirm a clinical diagnosis [6]. Diverticulitis can be further subdivided into uncomplicated or complicated diverticulitis. Uncomplicated diverticulitis usually is treated conservatively with the use of food and drink restriction, antibiotics, and/or fluid resuscitation. Complicated diverticulitis, however, can result in extended hospitalization times and possible surgical intervention [8].

About two-thirds of patients diagnosed with diverticulitis present with an uncomplicated disease course, while one-third of patients present with complicated diverticulitis [9]. The Hinchey classification is an example of a CT-based system used to classify acute diverticulitis in order to gauge the severity of a disease and determine treatment modalities. Following classification guidelines, patients deemed to have uncomplicated diverticulitis are staged 0-1. More complicated diseases, such as those that require treatment with antibiotics and commonly undergo elective resection, are classified as stage 2. The most severe diverticulitis are patients who scored within stages 3-4 as these cases need emergent surgical interventions and whose diverticulitis is likely purulent with fecal peritonitis [10]. In addition to intravenous antibiotics, as well as food and drink restriction, treatment for complicated diverticulitis may require surgical intervention [2]. When considering surgical options for the treatment of complicated diverticulitis, patient quality of life must also be taken into consideration. For example, one study found that, following the Hartmann procedure, 36.9% of patients were left with a permanent stoma in comparison to 8.9% of patients who instead underwent a resection and primary anastomosis [11]. Leaving a permanent stoma on a patient may lead to a decreased quality of life due to the constant presence of a colostomy bag.

As previously mentioned, diverticulitis was thought to be a disease seen commonly in geriatric populations. This characteristic patient population, however, is decreasing in age, and diverticulitis is now being diagnosed in younger patients. Previously admitted patients with acute diverticulitis in those under 50 years old ranged from 2 to 7%; that number has now almost increased fivefold, with percentages as high as 18%-34% [7,12]. Another study reported that, out of 238 patients over a nine-year period, 26% were under the age of 40, with an average age of 32 [6]. In the population of young patients with diverticulitis, it is noted that men were affected more than women with a ratio of 3:1, which is consistent with that seen in older populations [13]. It has since been proven that, in comparison to older patients with diverticulitis, younger people do not have more complicated diseases or undergo more elective surgeries. Unfortunately, in comparison to older patients, there is a higher recurrence rate among younger patients [12].

Diverticulitis classically has been diagnosed in older populations, and thus guidelines are written based on the clinical outcomes of this specific population. As obesity and other lifestyle factors begin to become more prevalent in younger populations, we are diagnosing and treating younger patients with acute diverticulitis. The rise in younger patients with acute diverticulitis has been described in the literature as noted above, but still much is unknown. Therefore, the objectives of this study are to 1) investigate the demographics and risk factors associated with the development of acute diverticulitis in a patient population under the age of 40 and 2) to investigate the relationship between diverticulitis and treatment modalities in a patient population under the age of 40. With this information, we seek to be able to provide insight not only into prevention but to also open up a broad discussion as to the best treatment modalities for these young patients.

This article was previously presented as a meeting abstract at the 2022 ACOS Clinical Assembly on September 15, 2022.

## Materials and methods

Adult Institutional Review Board (IRB) approval (St. Vincent's Medical Center, approval number: SVMC 19-365) was received. A retrospective review of the electronic medical records of a cohort of subjects diagnosed with diverticulitis between the years 2008-2019 was performed. Inclusion criteria included patients aged 18-40 who were treated at a single urban medical center with a diagnosis of acute diverticulitis or diverticulitis with any complications, including abscess, fistula, stricture, or perforation. Demographic data collected included age, sex, height, weight, and past medical, surgical, and family history. Clinical data collected included radiographic imaging results, serological laboratory results, microbiology, and pathology data. A total of 135 patient charts were reviewed, and 109 patients met the inclusion criteria. Categorical variables are summarized with numbers and percentages.

Data analysis was performed using R (version 4.0.2; R Foundation for Statistical Computing, Vienna, Austria). To analyze descriptive statistics, quantitative variables were expressed as mean ± SD, standard error, and 95% confidence interval, if they had a normal distribution (by the Kolmogorov-Smirnov test). One-way analysis of variance (ANOVA) was used to assess the significance of individual predictive parameters on the resulting outcome variable (conservative therapy, emergent surgery, elective surgery). The effects of independent variables on outcome were further investigated with the ANOVA post-hoc test for multiple comparisons to differentiate the significance between dependent outcome groups. Results were considered to be significant at a P-value <0.05.

## Results

Study population characteristics

There was a total of 109 patients enrolled in the study, with 71.6% female and 28.4% male (Table 1). Race was divided into six categories: Black, Caucasian, Hispanic, Asian, Other, and Unknown. The majority of patients were Caucasian at 39.4%, followed by Hispanic at 34.9%; race was unknown in 5.5% of participants (Table 1). The majority of participants (54.1%) did not have a first-degree relative diagnosed with diverticulitis; however, 23.9% of patient’s relative family history was unknown. The ages ranged from 19 to 40, with an average of 34 years old. The BMI of the patients ranged from 19.7 to 55, with a mean BMI of 34.5.

**Table 1:** Demographic characteristics.

Characteristics	Description	Percentage
Gender	Male	28.4%
	Female	71.6%
Race	Black	16.5%
	White	39.4%
	Hispanic	34.9%
	Asian	1.8%
	Other	0.9%
	Unknown	5.5%
Family History	None	54.1%
	1st degree relative	20.2%
	Unknown	23.9%
	Positive, relative degree unspecified	1.8%

Lifestyle characteristics

The average alcohol consumption per week was stratified by either greater than or less than four drinks a week. The majority of patients either did not consume alcohol (31.2%) or consumed less than four drinks per week (45.9); only a small subset of patients consumed more than four drinks per week on average (14.7%) (Table 2). Tobacco use was separated into never-used, current smokers, and former smokers. The majority of patients did not use tobacco (52.3%) (Table 2). In participants that did use tobacco, the majority were current users, smoking less than one pack per day (21.1%). Most patients were found to not have a history of drug use (59.6%); however, a large portion of patients’ drug use history was unknown (24.8%) (Table 2).

**Table 2:** Lifestyle characteristics. Alcohol use measured by drinks per week. Coffee was measured by cups per day. PPD = packs per day

Lifestyle habit	Description	Percentage
Alcohol	No use	31.2%
	<4 drinks	45.9%
	>4 drinks	14.7%
	Unknown	8.3%
Tobacco	No use	52.3%
	<1 PPD	21.1%
	>1 PPD	9.2%
	Former use <1PPD	0.9%
	Former use >1PPD	3.7%
	Former/Unknown	12.8%
Coffee	No use	2.8%
	>3 cups	2.8%
	Unknown	94.5%
Drugs	No	59.6%
	Yes	14.7%
	Former use	0.9%
	Unknown	24.8%
High Fat Diet	No	1.8%
	Unknown	98.2%
Low Fiber Diet	Unknown	100.0%

Diverticulitis complications

Diverticulitis complications were divided into four categories: abscess, perforation, fistula, or stricture. Thirteen patients (11.9%) presented with more than one diverticulitis complication, all including abscess and perforation. More than one complication is defined here as a fistula or stricture in addition to an abscess or perforation. Management types were divided into conservative, emergent surgery, and elective surgery. A one-way ANOVA and an individual post hoc analysis revealed that the following variables had significant differences in diverticulitis management type: the presence of any complication (p=0.015), Hinchey classification (p<0.001), and the individual complications of perforation (p=0.022), fistula (p=0.023), and acute abdomen (p=0.017) (Table 3). There was no significant difference between management type for abscesses (p=0.406) or strictures (p=0.808) (Table 3), elevated white count (p=0.636), or symptoms of nausea (p=0.473) or diarrhea (p=0.337). Further, there was no significant difference in conservative management vs. elective surgery for any complication.

**Table 3:** Diverticulitis complications. Complications seen needing conservative therapy, emergent surgery, or elective surgery. All complications were present before medical intervention.

Complications	Percentage	Overall significance	Conservative vs. emergent	Emergent vs. elective
Complicated		p=0.015	p=0.004	p=0.013
Diverticulitis				
Yes	35.8%			
No	64.2%			
Hinchey scale		P<0.001	P<0.001	P<0.001
1	82.6%			
2	16.5%			
3	0.9%			
Abscess		p=0.406		
Yes	18.3%			
No	81.7%			
Perforation		p=0.022	p=0.006	p=0.01
Yes	26.6%			
No	73.4%			
Fistula		p=0.023	p=0.007	p=0.043
Yes	1.8%			
No	98.2%			
Stricture		p=0.808	-	-
Yes	1.8%			
No	98.2%			
Acute abdomen		p=0.017	p=0.009	p=0.005
Yes	11.1%			
No	88.9%			
Location		-	-	-
Sigmoid	78.9%			
Descending	13.8%			
Transverse	1.8%			
Ascending	0.9%			
Cecum	4.6%			

Medical management - demographic characteristics and symptomology

After extensive chart review, we found that, of the population of 109 patients, 40 required surgical intervention (33 electives, seven emergent), and 69 patients were managed conservatively. Utilizing a one-way ANOVA and individual ad-hoc analysis, we found that the following variables had significant differences in the resulting diverticulitis management type: Hinchey classification, any complication, perforation and fistula, and acute abdomen. There was no significant difference, however, between management types for abscesses or strictures. Additionally, using the Hinchey classification to stratify risk was the single best predictor for management type. Within our study, 82.6% of patients were in Hinchey scale 1, 16.5% in Hinchey scale 2, and 0.9% in Hinchey scale 3. Therefore, as current management guidelines suggest, the Hinchey classification should continue to be used as a variable when deciding treatment options for patients under the age of 40.

Medical management was first divided into either conservative management or surgical management. The demographic characteristics of age (p=0.541), gender (p=0.713), and race (p=0.471) were not found to be significantly different between management groups (Table 4). Medical histories, such as BMI (p=0.377), diabetes mellitus (p=0.433), and irritable bowel disease (p=1.0), were not significantly different between management groups. Medical history of hypertension (p=0.078) was also not significantly different between groups. First-time diagnosis of diverticulitis (p=0.002) and Hinchey classification (p=0.030) were found to be significantly different between management groups. No symptoms upon presentation, such as abdominal pain location (p=0.494), constipation (p=0.212), or diarrhea (p=0.291), were found to be significantly different between the choices of management.

**Table 4:** Relationship between demographic characteristics and symptom presentation in regards to treatment with conservative vs surgical management. SD = standard deviation; BMI = body mass index; DM = diabetes mellitus; HTN = hypertension; IBD = irritable bowel disease; 1st diagnosis = first time patient was diagnosed with diverticulitis; LLQ = left lower quadrant; RLQ = right lower quadrant

Characteristics	Description	Conservative management (n=69)	Elective surgery or emergency surgery (n=40)	P-value
Mean sge (SD)	(Years)	33.72 (4.28)	34.24 (4.38)	p=0.427
Gender	Male	18	13	p=0.621
	Female	51	27	
Race	Black	11	7	p=0.512
	White	26	17	
	Hispanic	26	12	
	Asian	2	0	
	Other	4	3	
Mean BMI (SD)	kg/m^2^	34.00 (7.09)	35.56 (7.91)	p=0.332
DM	Yes	5	3	p=0.417
	No	64	36	
HTN	Yes	9	1	p=0.083
	No	60	39	
IBD	Yes	3	2	p=1.0
	No	66	38	
1st Diagnosis	Yes	14	19	p=0.0057
	No	55	21	
Abdominal pain	LLQ	48	26	p=0.471
	RLQ	8	2	
	Suprapubic	2	3	
	Diffuse	7	7	
	Other	4	2	
Constipation	Yes	15	33	p=0.213
	No	54	7	
Diarrhea	Yes	24	10	p=0.227
	No	45	30	
Hinchey	1	62	28	p=0.030
	2	7	11	
	3	0	1	

Medical management groups were further divided into one of three groups: conservative management, elective surgery, or emergent surgery at the time of presentation (Table 5). Demographic characteristics of age (p=0.483) and gender (p=0.388) were found to not significantly differ between groups. Race (p=0.056) was found to be marginally insignificant between management types. There was no significant difference in BMI (p=0.604), diabetes mellitus (p=0.546), hypertension (p=0.228), and irritable bowel disease (p=0.428) between management groups. Like that seen in Table 4, the first-time diagnosis of diverticulitis (p=0.013) and all Hinchey classifications (p<0.001) were found to be significantly different between management groups. However, when further defining what type of surgery was conducted, constipation (p<0.001) and diarrhea (p<0.001) were found to significantly differ between management groups. Abdominal pain location (p=0.375) was still found to not significantly differ between management groups.

**Table 5:** Relationship between demographic characteristics and symptom presentation in regards to conservative management, elective surgery, or emergency surgery. SD = standard deviation; BMI = body mass index; DM = diabetes mellitus; HTN = hypertension; IBD = irritable bowel disease; 1st diagnosis = first time patient was diagnosed with diverticulitis; LLQ = left lower quadrant; RLQ = right lower quadrant

Characteristics	Description	Conservative management (n=69)	Elective surgery (n=33)	Emergent surgery (n=7)	P-value
Mean age (SD)	(Years)	33.67 (4.26)	34.64 (4.33)	33.0 (4.69)	p=0.483
Gender	Male	18	12	1	p=0.388
	Female	51	21	6	
Race	Black	11	6	1	p=0.056
	White	26	14	3	
	Hispanic	26	11	1	
	Asian	2	0	0	
	Other	0	1	0	
Mean BMI (SD)	kg/m^2^	34.00 (7.09)	35.73 (8.27)	34.82 (6.65)	p=0.604
DM	Yes	5	4	0	p=0.546
	No	64	29	7	
HTN	Yes	9	2	0	p=0.228
	No	60	31	7	
IBD	Yes	3	1	1	p=0.428
	No	66	32	6	
1st Diagnosis	Yes	14	18	1	p=0.013
	No	55	15	6	
Abdominal pain	LLQ	48	22	4	p=0.375
	RLQ	8	1	1	
	Suprapubic	2	2	1	
	Diffuse	7	7	0	
	Other	4	1	1	
Constipation	Yes	15	6	0	p<0.001
	No	54	27	7	
Diarrhea	Yes	22	10	0	p<0.001
	No	45	23	7	
Hinchey	1	62	26	2	p<0.001
	2	7	7	4	p=0.004
	3	0	0	1	p<0.001

## Discussion

The Hinchey classification has been shown in various studies to accurately measure the risk of diverticulitis, with higher scores being associated with increased length of hospitalization and increased incidence and severity of complications [10]. Our data suggest that the Hinchey classification to stratify risk was the most successful predictor for management type for patients under the age of 40 (Table 3). Additionally, the Hinchey classification was not only significantly different between conservative management and surgical intervention but also when further specifying when to perform surgery (Tables 4-5). Therefore, the Hinchey classification can be used not only to decide whether a patient’s condition warrants surgery but also whether emergent or elective surgery is necessary. Additionally, the staging results could be utilized to guide treatment management in younger populations in a similar manner to the Hinchey classification, which is currently functioning in older patient populations [10].

Diverticulitis complications were also shown to be statistically significant when stratified by medical management. Our study divided complications into four categories: abscess, perforation, fistula, and stricture. Interestingly, 35.8% of our study population presented with complicated diverticulitis. This value is much larger than other studies that show an average population of complicated diverticulitis at about 8.9-10% [14]. Our study may suggest that the higher incidence of complications may be related to the younger age of our patient population. Therefore, the current treatment algorithm relating diverticulitis complications with only surgical intervention may not be appropriate for patients under the age of 40 as previously thought. As previously discussed, our data further suggest that patients presenting with abscesses or strictures may not need surgical intervention. These findings should be considered when evaluating patients under the age of 40 and taken into account in comparison with current guidelines. Furthermore, the presence of a fistula was only marginally significant when comparing elective to emergent surgery (Table 3). Understanding the pattern of complication presentation and successful medical management in this younger population can help guide treatment algorithms that can help prevent unnecessary intervention. This differentiation is vital in the process of developing treatment guidelines for diverticulitis in patients under 40 years, as the common treatments for complicated vs uncomplicated diverticulitis vary drastically in terms of invasiveness, hospitalization length-of-stay, and cost of treatment [15].

We did not find that patient demographics, when analyzed in relation to treatment modality, were statistically significant. This contrasts with a study conducted in 2021 that reported a difference in race among Medicare diverticulitis patients in terms of treatment choices with or without ostomy [16]. When considering medical history, our study showed that a first-time diagnosis of diverticulitis was more likely to be managed surgically, whereas patients with recurrent diverticulitis were more likely to be treated conservatively. This contrasts with a recent review of over 30,000 cases, which showed that more patients were treated conservatively than with emergent surgery regardless of primary or recurrent diagnosis [17]. However, their research did showcase how hospital readmission following diverticulitis was more common in patients treated conservatively when compared to those treated surgically. Additionally, our analysis showed that, in relation to symptomatology, the Hinchey score was the most significant predictor of the treatment modality. Patients scoring a 1 in the Hinchey classification were more likely to be managed conservatively, while patients scoring a 2 or 3 were more likely to be managed surgically. However, there is a limitation to this result as only one patient out of our cohort scored a Hinchey score of 3. Therefore, further research should be conducted to determine its significance. This pattern of treatment is contradictory to that seen in a case-control study conducted by Brandt et al., which states that emergency surgery for Hinchey type II diverticulitis should be avoided due to its high morbidity and mortality rate [18]. Therefore, our data argue that younger patients with a Hinchey score of 2 may be more amenable to surgery as compared to conservative treatment alone.

Our study divided complications into four categories: abscess, perforation, fistula, and stricture. Of these complications, 18.3% had an abscess, 26.6% had perforation, 1.8% had a fistula, 1.8% had strictures, and 11.1% had an acute abdomen. All complications seen were deemed to undergo either conservative therapy, emergent surgery, or elective surgery. This was similar to previous studies, which stated that surgical resection is the common treatment for complicated and recurrent diverticulitis. However, our data state that these younger patients with elevated white count, certain abscesses, or stricture formation may not need emergent surgery at the time of presentation. Existing guidelines do not provide detailed additional information as surgical intervention in complicated diverticulitis is highly individualized and variable per each unique patient diagnosis [2]. Operative intervention in complicated diverticulitis is an ongoing debate, and additional information should be gathered for all ages, including the patients in our study under the age of 40.

Our data should be interpreted considering some limitations. Our research is also limited because of its retrospective nature, which allows for the occurrence of potential biases. Our findings are analyzed through the view of diverticulitis in patients under the age of 40, but we recognize that there are possible factors that we may have inadvertently overlooked that exist within this population such as the impact of education level, income, and sexual orientation as risk factors. Future research should focus on whether the location of resection in primary diverticulitis is associated with the risk of recurrence of diverticulitis as these factors were not tested within this dataset. Further analysis should also examine the utility of the individual levels of the Hinchey classification to predict outcomes in its relationship with recurrence. Future studies could be prospective in nature so that possible confounding factors and biases could be more accurately accounted for.

## Conclusions

Our study has found that a higher incidence of complications in diverticulitis may be related to the younger age of the patient. Additionally, when considering the pattern of complication presentation in younger patients with complicated diverticulitis, surgical intervention might not be appropriate. Clinically, the current treatment algorithm relates diverticulitis complications with surgical interventions. However, our data suggest that patients under the age of 40 presenting with abscesses or strictures may not need surgical intervention. This information could be particularly helpful in guiding physicians and younger patients in selecting the best choice of care and minimizing complications. Additionally, further research should help guide treatment protocol in this specific population of patients as there is a lack of established guidelines pertaining to diverticulitis surrounding younger patients.
